# Hainan mantle plume produced late Cenozoic basaltic rocks in Thailand, Southeast Asia

**DOI:** 10.1038/s41598-018-20712-7

**Published:** 2018-02-08

**Authors:** Quanshu Yan, Xuefa Shi, Ian Metcalfe, Shengfa Liu, Taoyu Xu, Narumol Kornkanitnan, Thanyapat Sirichaiseth, Long Yuan, Ying Zhang, Hui Zhang

**Affiliations:** 1 Key Laboratory of Marine Sedimentology and Environmental Geology, First Institute of Oceanography, SOA, Qingdao, 266061 China; 2 Laboratory for Marine Geology, Qingdao National Laboratory for Marine Science and Technology, Qingdao, 266061 China; 3 School of Environmental and Rural Science, University of New England, Armidale, NSW 2351 Australia; 4 Marine and Coastal Resources Research and Development Center Upper Gulf of Thailand, 120/1 Bangyapraek, Meuang, Samut sakhon, 74000 Thailand

## Abstract

Intraplate volcanism initiated shortly after the cessation of Cenozoic seafloor spreading in the South China Sea (SCS) region, but the full extent of its influence on the Indochina block has not been well constrained. Here we present major and trace element data and Sr-Nd-Pb-Hf isotope ratios of late Cenozoic basaltic lavas from the Khorat plateau and some volcanic centers in the Paleozoic Sukhothai arc terrane in Thailand. These volcanic rocks are mainly trachybasalts and basaltic trachyandesites. Trace element patterns and Sr-Nd-Pb-Hf isotopic compositions show that these alkaline volcanic lavas exhibit oceanic island basalt (OIB)-like characteristics with enrichments in both large-ion lithophile elements (LILE) and high field strength elements (HFSEs). Their mantle source is a mixture between a depleted Indian MORB-type mantle and an enriched mantle type 2 (EMII). We suggest that the post-spreading intraplate volcanism in the SCS region was induced by a Hainan mantle plume which spread westwards to the Paleozoic Sukhothai arc terrane.

## Introduction

**Figure 1:**
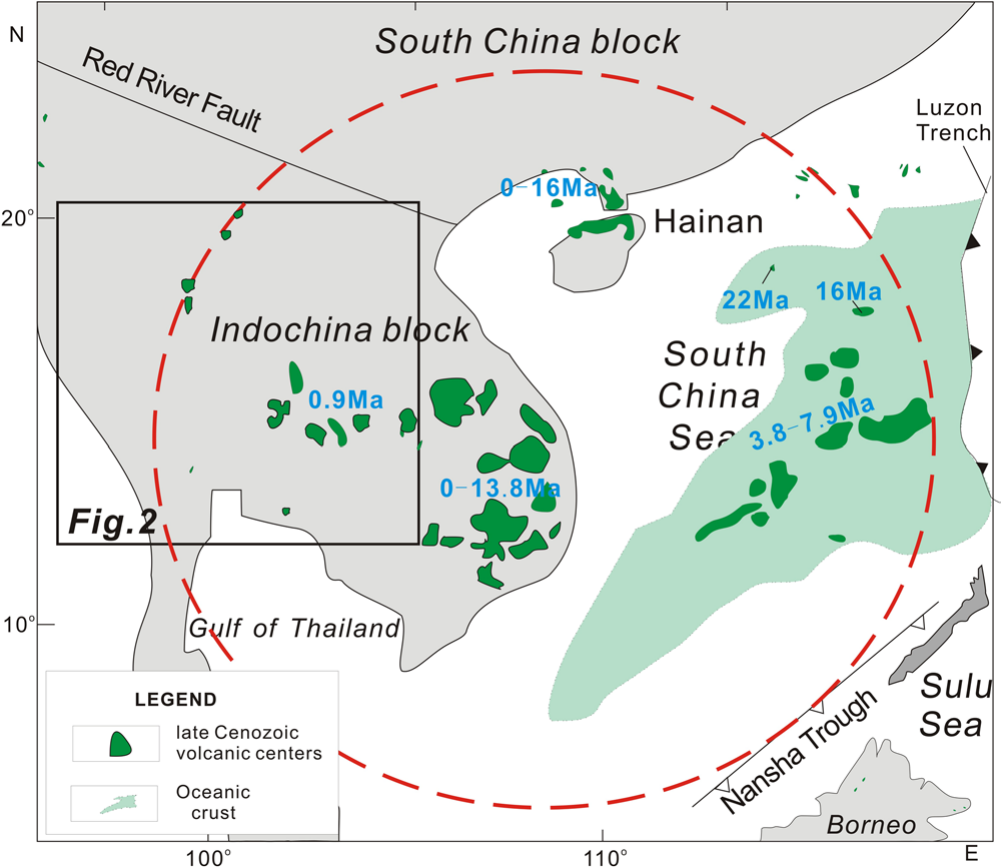
Distribution of late Cenozoic (<16 Ma) intraplate volcanism in the South China Sea region, which includes Beibuwan, Leiqiong peninsula, Pearl River mouth basin, SCS basin and the Indochina block^29^. Late Cenozoic volcanic rocks are mainly distributed within the red circle (deshed line), and include the Leizhou peninsula, Hainan Island, Pearl River Mouth Basin, Beibu gulf, South China Sea Basin, and Indochina block (reviewed by Yan *et al*.^29^), and their approximate ages are also shown. Detailed ages for late Cenozoic basalts are given in Fig. 2.

After the cessation of Cenozoic seafloor spreading (32–16 Ma)^1–4^ of the South China Sea, intraplate volcanism almost simultaneously affected large areas in the South China Sea region, e.g., the Pearl River Mouth Basin (PRMB)^5^, Leiqiong Peninsula^6–11^ and the Beibu Gulf^12–14^ in the northern margin of the SCS, the Indochina Block^15–25^, the Reed Bank and Dangerous Grounds^26,27^, and the SCS basin itself^27–30^ (Fig. 1). The OIB-like geochemical characteristics of the volcanic rocks are distinct from those associated with Late Cenozoic subduction related lavas from the Luzon arc east of the Manila trench^31,32^ and northeast Borneo (southeast of the Nansha trough/NW Palawan trough)^29,33^ (Fig. 1). Thus, the Manila trench and Nansha trough can be considered as eastern and southern boundaries, respectively, of the region affected by the intraplate volcanism^34^. However, the western extent of the intraplate magmatism is uncertain, as Tengchong^35^, Myanmar^36^ and possibly the western part of Thailand have been continuously affected by the subduction of the Indian plate beneath the Eurasian plate during the Cenozoic Era. Therefore, the geodynamic settings of several late Cenozoic volcanic centers in western Thailand need to be better constrained.

The geodynamic setting of the intraplate volcanism in the SCS region is still debated. Far field effects of the India-Asia collision may not only play a significant role on the Cenozoic tectonic evolution of the SCS region (e.g., the opening of the SCS)^2,37^, but also facilitate the upwelling of a Hainan mantle plume^29,34^. Moreover, the Hainan mantle plume has been invoked to account for the intraplate volcanism along the northern margin of the SCS^9,10^^,^^12–14^, in southern Vietnam^25^ and in the SCS basin^28–30^. Similar to other localities in the SCS region, the late Cenozoic intraplate volcanism of the Indochina block has also been suggested to have formed in an extensional tectonic settin^15–25^. Previous studies of Indochina Block Cenozoic volcanism indicate that the mantle source can be largely explained by a two mantle end-members mixing model, involving depleted Indian MORB-type mantle and enriched mantle type II (EMII)^20,23,25^. For the origin of EMII, early studies proposed an origin from the sub-continental lithospheric mantle (SCLM)^7,20,28^, but recently several studies on late Cenozoic volcanism in the SCS region related the enriched component to a mantle plume^9–11,25,28,30,38^. In order to clarify the geodynamic setting of Indochina block Cenozoic volcanism, we obtained new Hafnium isotope data as well as major- and trace element and Sr-Nd-Pb isotope ratios of late Cenozoic volcanic lavas from Thailand (Indochina block). These data are combined with other published data for the Indochina block and the whole SCS region and are used to constrain the petrogenesis and mantle source nature of late Cenozoic volcanic lavas from Thailand and their deep mantle geodynamic process.

## Geological setting and sampling details

**Figure 2:**
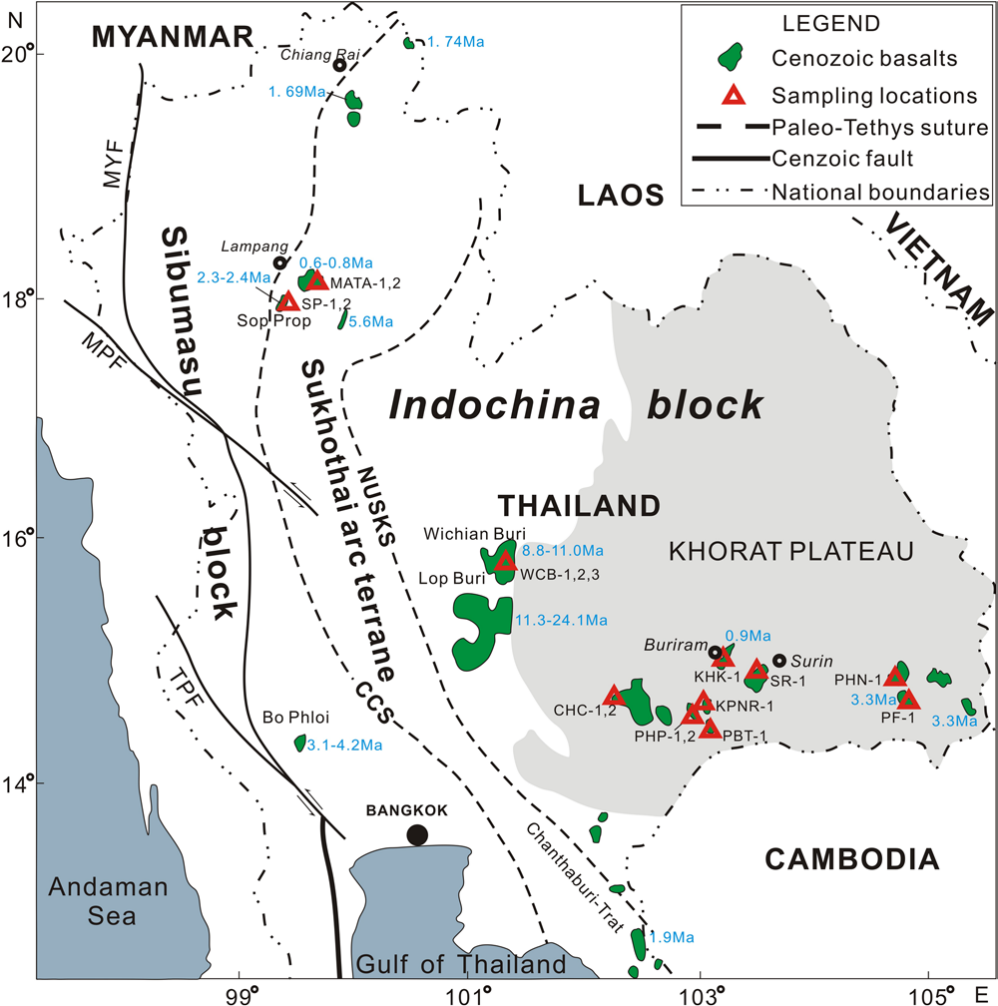
Schetch map of Thailand and surrounding regions showing the late Cenozoic volcanic centers with ages^23,41,43,44^, principal Cenozoic faults and Paleo-Tethys sutures^39^, national boundaries and sampling locations. MYF, Mae Yuam fault; MPF, Mae Ping fault; TPF, Three Pagodas fault; CCS, Chiangmai-Changthaburi suture; NUSKS, Nan-Uttaradit Sra Kaeo suture.

Thailand and the surrounding region can be divided into three tectonostratigraphic units: a western Sibumasu block (Sino- Burma- Malaya- Sumatra), a middle Sukhothai arc terrane and an eastern Indochina block hosting the Khorat Plateau. These three terranes are separated by two Paleo-Tethys sutures. The western suture is the Chiangmai-Chanthaburi suture and includes Middle Devonian to Middle Triassic radiolarian cherts and deep oceanic sediments. The other suture is the back-arc Nan-Uttaradit Sra Kaeo suture which is composed of disaggregated Paleozoic ophiolites and melanges^17,39,40^ (Fig. 2). Both the Sibumasu block and the Indochina block have Precambrian basements, and were part of the India–Australian margin of eastern Gondwanaland in the Early Paleozoic. These two blocks, together with the SCS region, have been affected by the Tethys tectonic regime during the late Paleozoic to early Mesozoic period, and subsequently by the Pacific ocean tectonic regime during the late Mesozoic^39–42^. In addition, two major Cenozoic strike-slip faults (Mae Ping fault and Three Pagodas fault) cut through the western part of Thailand (Fig. 2).

The seventeen volcanic rock samples of this study were collected from 11 basaltic flows close to the Chiangmai-Changthaburi suture and within and around the Khorat Plateau^23,43,44^ (Fig. 2). From two basaltic flows close to the Chiangmai-Changthaburi suture, we collected two basaltic rock samples (with phenocrysts of olivine (Fo = 82.9–84.5, clinopyroxene (Wo = 48.7, En = 40.0, Fs = 11.2) and plagioclase (An = 58.8–60.6)) from small outcrops in the Sop Prap basaltic flow (2.3–2.4 Ma) that covers an area of 70 km^2^, and two basalt samples (with phenocrysts of olivine (Fo = 83.3–90.7) and plagioclase (58.2–66.4)) from small outcrops in the Mae Tha basaltic flow (0.6–0.8 Ma) that extends over 120 km^2^ (Supplementary Dataset Table 1). From the western margin of the Khorat Plateau, we collected three basalt samples (with olivine (Fo = 76.2–81.8) and plagioclase phenocrysts (An = 56.1–60.6) and microphenocrysts) from the Wichian Buri basaltic flow (8.8–11.0 Ma) that covers an area of 200 km^2^ (Supplementary Dataset Table 1). Twelve samples were collected from small outcrops of eight dispersed basaltic flows within the Khorat Plateau including Na Khon Ratchasima (1400 km^2^, 0.9 Ma), Khao Kradong (120 km^2^, 0.9 Ma), Surin (55 km^2^, 0.9 Ma), Phu Naoan (23 km^2^, 3.3 Ma), Si Sa Ket (74 km^2^, 3.3 Ma), Khao Pha Nom Rong (20 km^2^, 0.9 Ma), Prai Bat (6 km^2^, 0.9 Ma), and Phu Phra (90 km^2^, 0.9 Ma) (Supplementary Dataset Table 1). The rocks collected from the plateau show porphyritic textures and contain sparse phenocrysts of olivine (Fo = 60.1–84.6), clinopyroxene (Wo = 39.7–45.7, En = 41.9–45.6, Fs = 9.0–13.9) and plagioclase (An = 46.5–63.8), and some microphenocrysts in the groundmass. The ages of the volcanic rocks range from 0.4 to 11 Ma^23,43,44^, and these samples can be distinguished into two groups by their ages, one is relatively older basalts with ages of 8.8–11.0 Ma, the other is younger ones with ages younger than 3.3 Ma (Supplementary Dataset Table 1).

## Analytical methods

For our study, a volume of 10–25 cm^3^ of basaltic samples was trimmed of vein fillings and alteration rinds in order to obtain the freshest material. The samples were leached in 4 N nitric acid for 3 hours to remove surface contamination, and crushed into 0.5–1 cm^3^ chips in a stainless steel mortar and pestle, rinsed in distilled water and dried twice. Several of the freshest chips from the interior of each sample were separated for Pb isotopic analysis. The remainders were powdered in an alumina ceramic shatterbox.

### Major and trace element analytical methods

Major elements for all samples were determined by X-ray fluorescence (XRF) spectroscopy at the Testing Center of Shandong Bureau, China Metallurgical Geology Bureau (TC-SB-CMGB). Samples powders were fused with lithium metaborate-lithium tetraborate, which also includes an oxidizing agent (lithium nitrate), and then poured into a platinum mould. The resultant disk was analyzed by the XRF spectroscopy. Loss on ignition (LOI) of samples was measured at 1050 °C, and after drying at 100 °C. Trace element compositions were measured using an inductively coupled plasma-mass spectrometer (ICP-MS), also at the TC-SB-CMGB. The precision of the XRF is ±0.2% to 2% for major elements present in concentrations >1 wt% (SiO_2_, Al_2_O_3_, and CaO) and about ±2% to 5% for minor elements present in concentrations <1.0 wt% (MnO, K_2_O, TiO_2_, and P_2_O_5_). The accuracy of ICP-MS for trace elements is better than 10%. The international standard sample (BHVO-2) was used to monitor drift during XRF and ICP-MS measurements, and was found to be consistent with the recommended values within the errors of the methods (Supplementary Dataset Table 1).

### Sr-Nd-Pb-Hf analytical methods

Sr-Nd-Hf isotopic ratios were measured using Neptune Plus multicollector ICP-MS (MC-ICP-MS) at the State Key Laboratory of Isotope Geochemistry, Guangzhou Institute of Geochemistry, Chinese Academy of Sciences. The procedure for Sr-Nd-Hf isotopic analytical methods is the same as those described by Wei *et al*.^45^, Liang *et al*.^46^, and He *et al*.^47^. Normalizing factors used to correct the mass fractionation of Sr and Nd during the measurements are ^86^Sr/^88^Sr = 0.1194 and ^146^Nd/^144^Nd = 0.7219, respectively. The measured ^176^Hf/^177^Hf ratios were normalized to ^179^Hf/^177^Hf = 0.7325, and are reported adjusted relative to the standard JMC-475 with a ^176^Hf/^177^Hf = 0.282160. Reference standards were analyzed along with samples and give ^87^Sr/^86^Sr = 0.710266 ± 7 (2σ) for NBS987, ^143^Nd/^144^Nd = 0.512105 ± 6 (2σ) for JNdi-1^48^, and ^176^Hf/^177^Hf = 0.282186 ± 4 (2σ) for JMC475.

Pb isotopic ratios were measured using a High resolution (Nu Instruments Ltd, Wrexham, North Wales, UK) multi-collector inductively coupled plasma- mass spectrometer at the Key lab of Marine Sedimentary and Environmental Geology, State Oceanic Administration, China. Details of the Pb separation procedure are presented by Janney and Castillo^49^. The Pb standard NBS 981 was used to correct the measured isotopic ratios of samples for isotopic fractionation and the average correction was 0.1% per atomic mass unit. Procedural blanks were <0.5 ng for Pb. During the analysis, NBS981 standard yielded an average value of ^206^Pb/^204^Pb = 16.9382, ^207^Pb/^204^Pb = 15.4935, and ^208^Pb/^204^Pb = 36.7255.

## Analytical Results

### Major and trace element compositions

**Figure 3:**
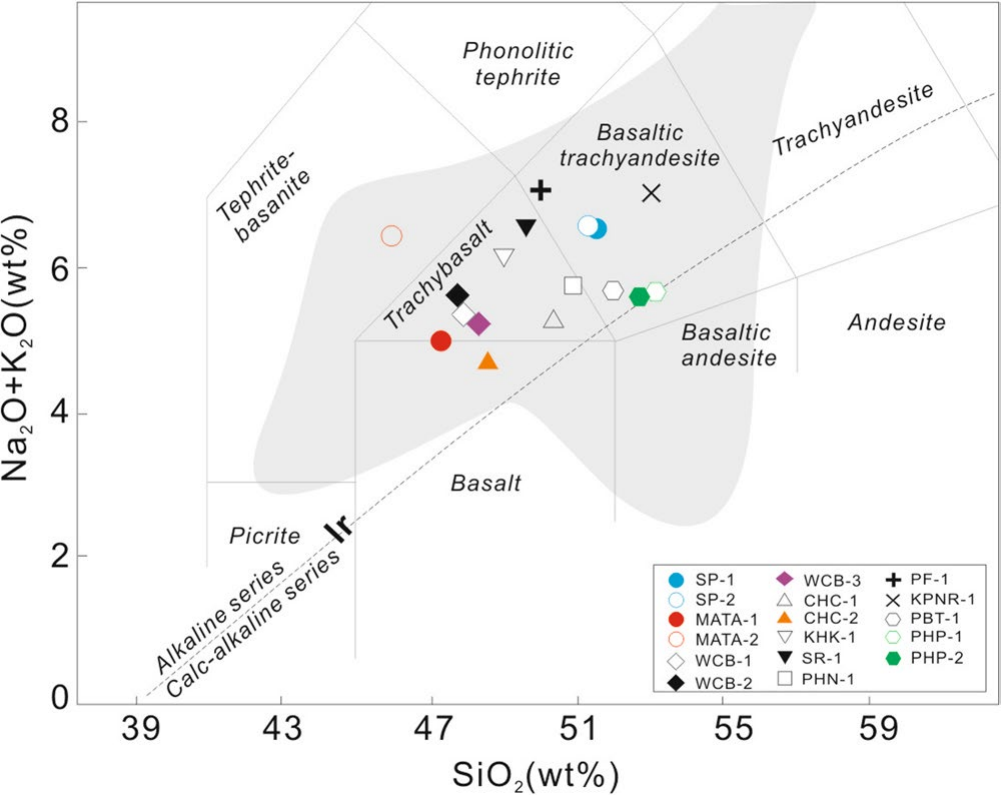
TAS (SiO_2_ vs. Na_2_O + K_2_O) and alkaline discrimination diagrams for late Cenozoic volcanic rocks from Thailand (Indochina block)^50^. Data field in grey is literature based for the Indochina block^20–25^.

Bulk rock major and trace element compositions are reported in Supplementary Dataset Table 1. Loss on ignition (LOI) values of the samples range from 0.16 to 2.71 wt. %, which are due to variable amounts of secondary hydrous/altered minerals. After major oxides analyses recalculated to 100% on an H_2_O and CO_2_− free (basically represented by LOI in this study) basis, all samples were plotted on a plot of total alkalis (Na_2_O + K_2_O) versus silica (SiO_2_)^50^ (Fig. 3). In Fig. 3, samples plot into the fields of trachybasalt, basaltic trachyandesite, basalt (CHC-2 from the Na Khon Ratchasima basaltic flow the Mae Tha basaltic flow) and basanite, and all other samples belong to the alkaline lava series (Fig. 3).

**Figure 4:**
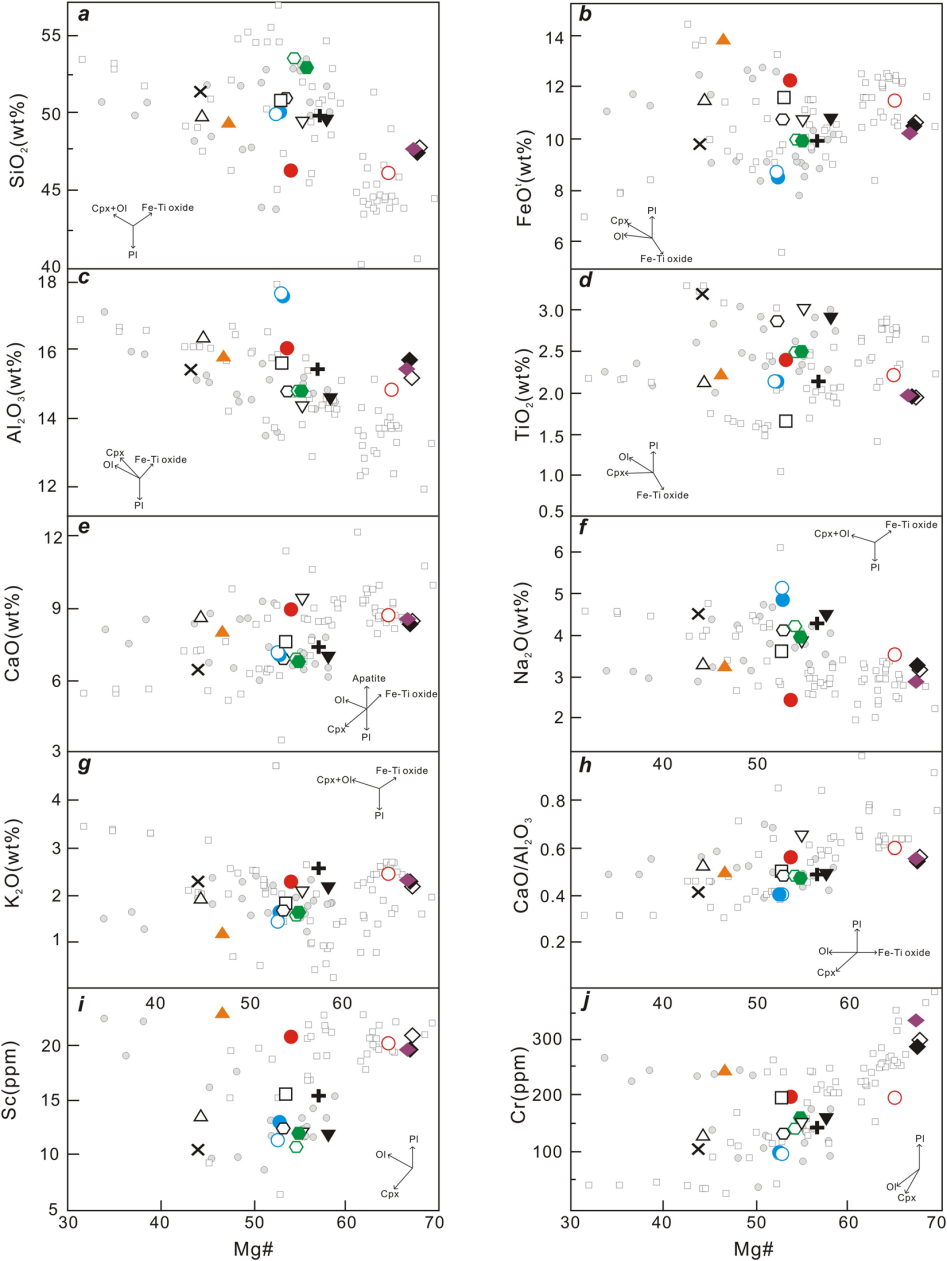
Variations of selected oxides, trace element and element ratios of late Cenozoic basalts from Thailand as functions of Mg^#^. Mg^#^ = 100 × Mg/(Mg + Fe), Fe^2+^/Fe^total^ = 0.90, cation ratio. Symbols are the same as Fig. 2, and small circles are data for basaltic rocks from the Indochina block. Other data sources are the same as Fig. 3.

The samples have a wide range in MgO (4.09–9.38 wt.%), with Mg# values of 43.9–67.7, and abundances of compatible trace elements (Ni = 58–240 ppm, Co = 26–50 ppm, Cr = 97–346 ppm) (Supplementary Dataset Table 1), that are too lower than those for partial melts from peridotite mantle (Mg# > 70, Ni > 400–500 ppm, Cr > 1000 ppm; see literature^51,52^. Relative to those younger samples, the older ones from Wichian Buri basaltic flow have highest MgO and Mg#. In the correlation diagrams of Mg# versus other oxides, CaO/Al_2_O_3_ and trace elements (Sc, Cr) decrease with decreasing Mg# to 43.9, and increasing SiO_2_ (Fig. 4a), FeO^t^ (Fig. 4b), Al_2_O_3_ (Fig. 4c), TiO_2_(Fig. 4d), and a decreasing trend in CaO/Al_2_O_3_(Fig. 4h), Sc (Fig. 4i) and Cr(Fig. 4j). There is no co-variation between Mg# and CaO, Na_2_O and K_2_O (Fig. 4e,g).

**Figure 5:**
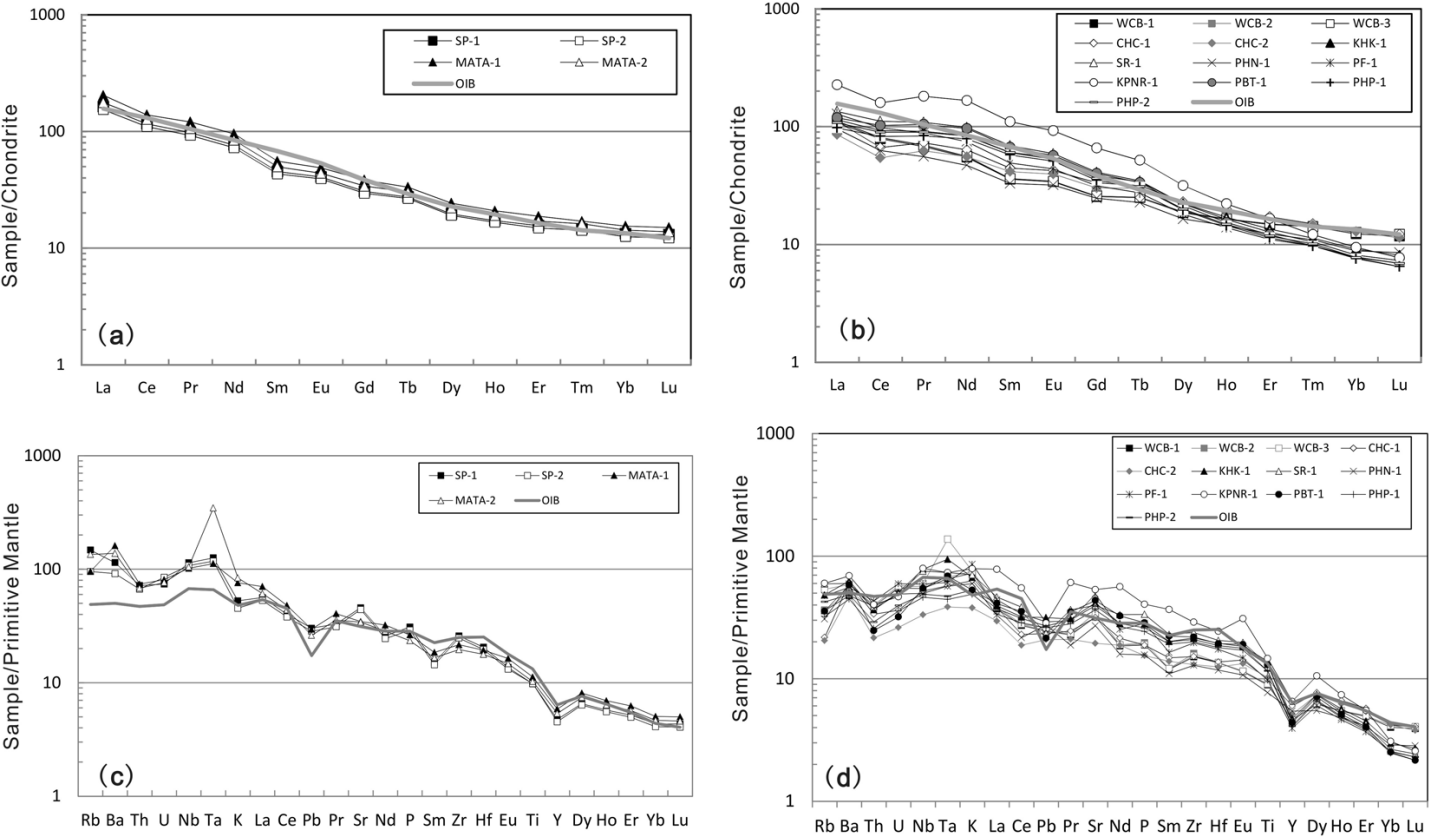
Chondrite-normalized rare earth element distribution patterns and primitive mantle-normalized trace element concentration diagram for late Cenozoic volcanic rocks from the Paleozoic Sukhothai arc terrane (**a,c**) and the Khorat plateau (**b,d**). Distribution patterns for compositions of oceanic island basalts (OIB) are also shown for reference. Trace element abundances of the primitive mantle (PM), Chondrite and OIB are from Sun and McDounough^53^.

Several samples from the Paleozoic Sukhothai arc terrane exhibit light rare earth element (LREE) enrichment ([La/Yb]_N_ = 12.2–13.2) with no Eu anomaly (Fig. 5a) and similar to those from the Kohrat plateau ([La/Yb]_N_ = 6.7–24.0) (Fig. 5b) on the plots of chondrite-normalized REE patterns. In primitive mantle-normalized trace element diagrams, samples from both the Paleozoic Sukhothai arc terrane and the Kohrat plateau are generally enriched in large ion lithophile elements (LILEs) and high field strength elements (HFSEs, e.g., positive Nb-Ta anomaly), although samples from the Sukhothai arc terrane have slightly higher concentrations of LILEs than those from the Kohrat plateau (Fig. 5c,d). In general, these alkaline lavas from Thailand are similar to those reported from previous studies on late Cenozoic volcanic rocks from Thailand and Vietnam^21,23,25^ and are typical oceanic island basalts (OIBs) with (La/Yb)N = 12.3^53,54^.

### Sr-Nd-Hf-Pb isotopic compositions

**Figure 6:**
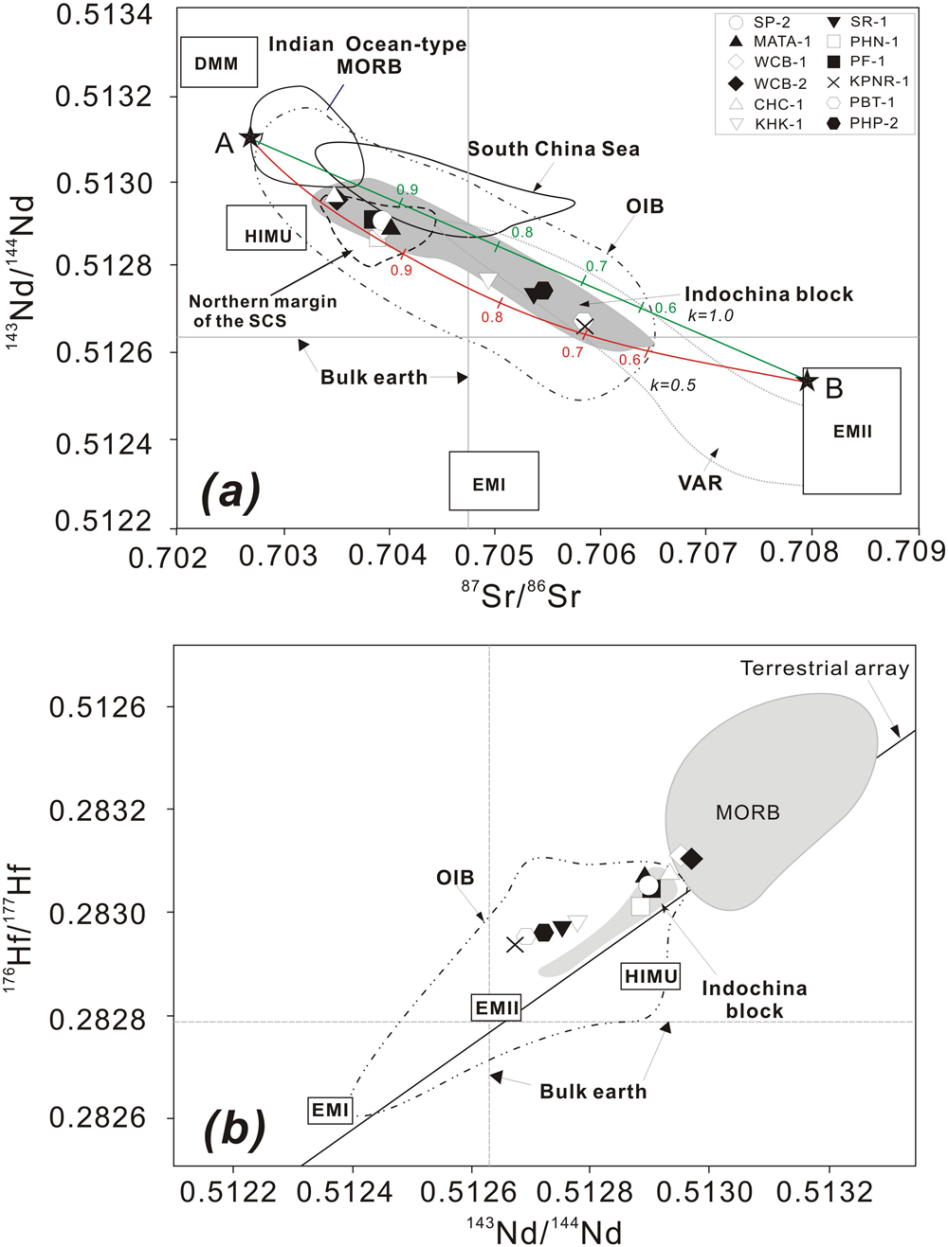
(**a**) ^143^Nd/^144^Nd vs. ^87^Sr/^86^Sr and (**b**) ^176^Hf/^177^Hf vs. ^143^Nd/^144^Nd isotopic ratios of late Cenozoic volcanic rocks from Thailand. Data for northern margin of the SCS including Zhujiangkou basin, Beibu gulf, Niutoushan and Penghu basalts are from references^5,7,9,11,14,56,57^. Data for South China Sea are from the literature^27–30^. Data for Indochina block are from references^20,22–25^. Fields representing late Cenozoic volcanic arc rocks (VAR)^36^ from Tengchong, Linzizong, Myanmar and Andaman-Java in (**a**) are also shown for comparison. The approximate fields for DMM, HIMU, EM1, and EM2 are from references^89,90^, for OIB from Castillo^55^ and for Indian Ocean-type MORB from Mahoney *et al*.^91^. The approximate fields for MORB (EPR/Atlantic/Indian), HIMU, EM1, EM2, and OIB in (**b**) are from references^25,92^. The bulk earth ^176^Hf/^177^Hf and the terrestrial array from references^93–95^ in (**b**) were also shown. In Fig. 6a, modeling parameters for end-member mixing is as follows, A for a depleted end member^96^: Sr (ppm) = 7.66, Nd (ppm) = 0.58, ^87^Sr/^86^Sr = 0.7026, ^143^Nd/^144^Nd = 0.51311; B for an enriched end member^97^: ^87^Sr/^86^Sr = 0.7078, ^143^Nd/^144^Nd = 0.51258. Tick marks with numbers represent % contributions from the DMM to the mixture, and three lines/curves represent different *k* or Sr/Nd ratios. In general, isotopic compositions for late Cenozoic alkaline rocks from Thailand can be produced by adding about >60% depleted mantle melt to enriched melt. Errors (2σ) are smaller than the size of symbols.

**Figure 7:**
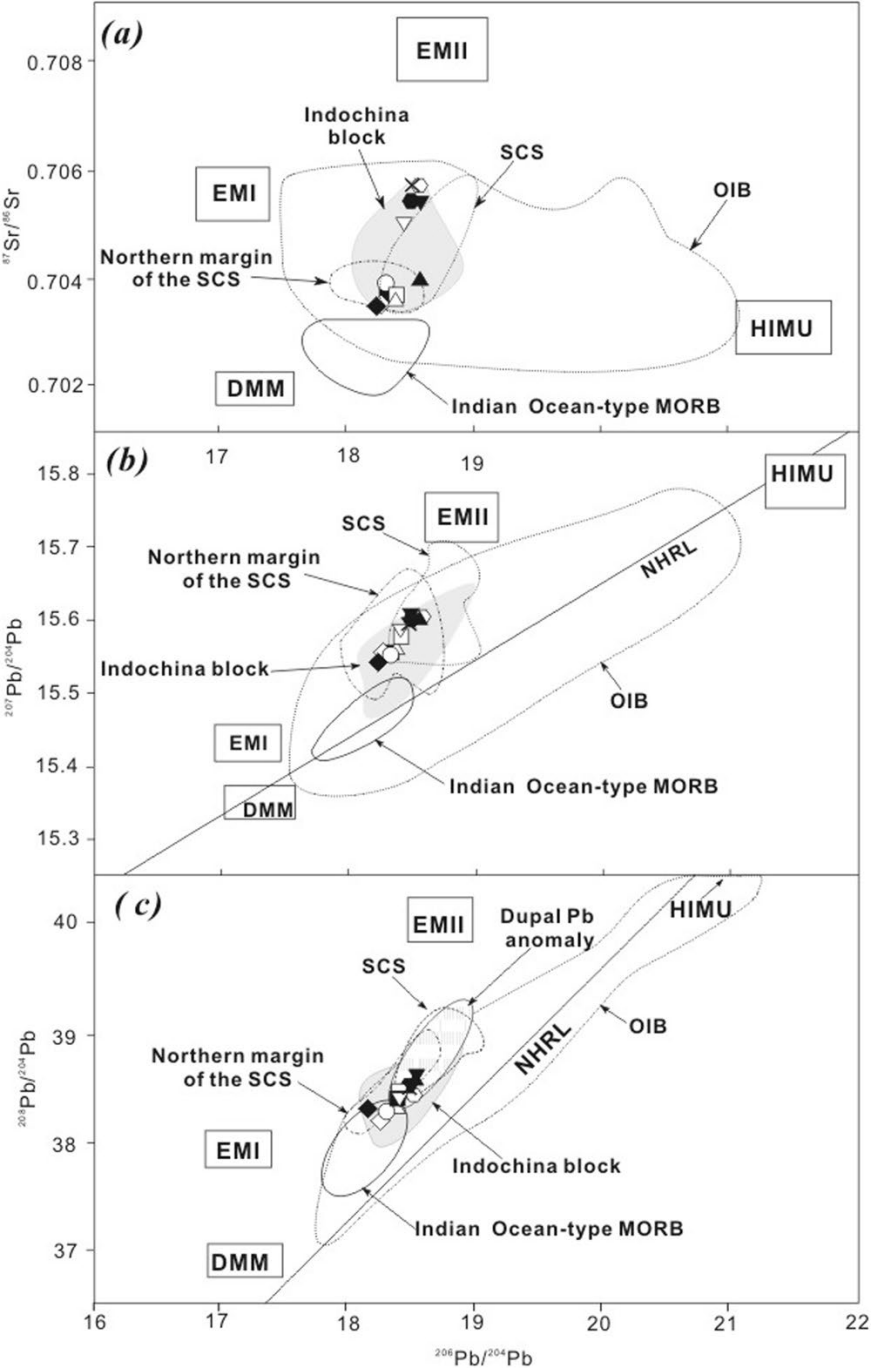
(**a**) ^87^Sr/^86^Sr versus ^206^Pb/^204^Pb, (**b**) ^208^Pb/^204^Pb versus ^206^Pb/^204^Pb, and (**c**) ^207^Pb/^204^Pb versus ^206^Pb/^204^Pb plots for the late Cenozoic volcanic rocks from Thailand. Data sources for the fields of DMM, HIMU, EM1, EM2, OIB, Indian Ocean-type MORB are the same as in Fig. 6a. Other data such as northern margin of SCS, SCS and Indochina block are also the same as Fig. 6a. The field for Dupal anomaly is from Hamelin and Allègre^98^, NHRL is North Hemisphere reference line^58^. Errors (2σ) are smaller than the size of symbols.

The Sr, Nd, Hf, and Pb isotopic compositions for the Thailand basaltic rocks are listed in Supplementary Dataset Table 2 and shown on several isotopic correlation diagrams (Figs 6 and 7). Age correction was not applied because the samples are relatively young (majority of them <3.3 Ma, Supplementary Dataset Table 1), and measured isotopic ratios can be regarded as their initial ratios (i.e., no effect from radiogenic ingrowth). The Sr, Nd, Hf and Pb isotopic ratios for these samples are as follows, ^87^Sr/^86^Sr = 0.70356–0.70588, ^143^Nd/^144^Nd = 0.51267–0.51297 (εNd =  + 1.7 to 5.7), ^176^Hf/^177^Hf = 0.28294–0.28312 (εHf =  + 4.0 to 10.9), ^206^Pb/^204^Pb = 18.204–18.582, ^207^Pb/^204^Pb = 15.540–15.613, and ^208^Pb/^204^Pb = 38.193–38.646 (Supplementary Dataset Table 2). In the ^143^Nd/^144^Nd versus ^87^Sr/^86^Sr correlation plot (Fig. 6a), all alkali samples from Thailand have consistent plots with data from previous studies on late Cenozoic basaltic rocks from the Indochina block^20–25^ and of OIB^55^. Moreover, the samples have slightly more variable Sr and Nd isotope ratios than samples from the northern margin of the SCS^5,7,9,11–14,29,56,57^. In the ^176^Hf/^177^Hf vs. ^143^Nd/^144^Nd diagram (Fig. 6b), all samples in this study plot in the field of OIB and cover a range that is slightly larger than that of Vietnamese basalts^25^. There is a positive correlation between Hf and Nd isotopes, suggesting that these two isotope systems are coupled. In the Sr–Pb, Pb–Pb diagrams, all alkali samples from Thailand also plot within the Indochina block and OIB fields (Fig. 7) as well as within or close to the field of the SCS itself and the northern margin of the SCS. The samples plot above and roughly subparallel to the Northern Hemisphere reference line (NHRL)^58^. In general, Sr, Nd, Hf, and Pb isotopic compositions for the alkali basaltic rocks from Thailand fall between the Indian ocean-type mantle or depleted MORB-type mantle (DMM) and an enriched mantle type II (EMII) (Figs 6 and 7).

## Discussion

### Petrogenesis of late Cenozoic basaltic rocks from Thailand

#### Possible continental crustal contamination?

**Figure 8:**
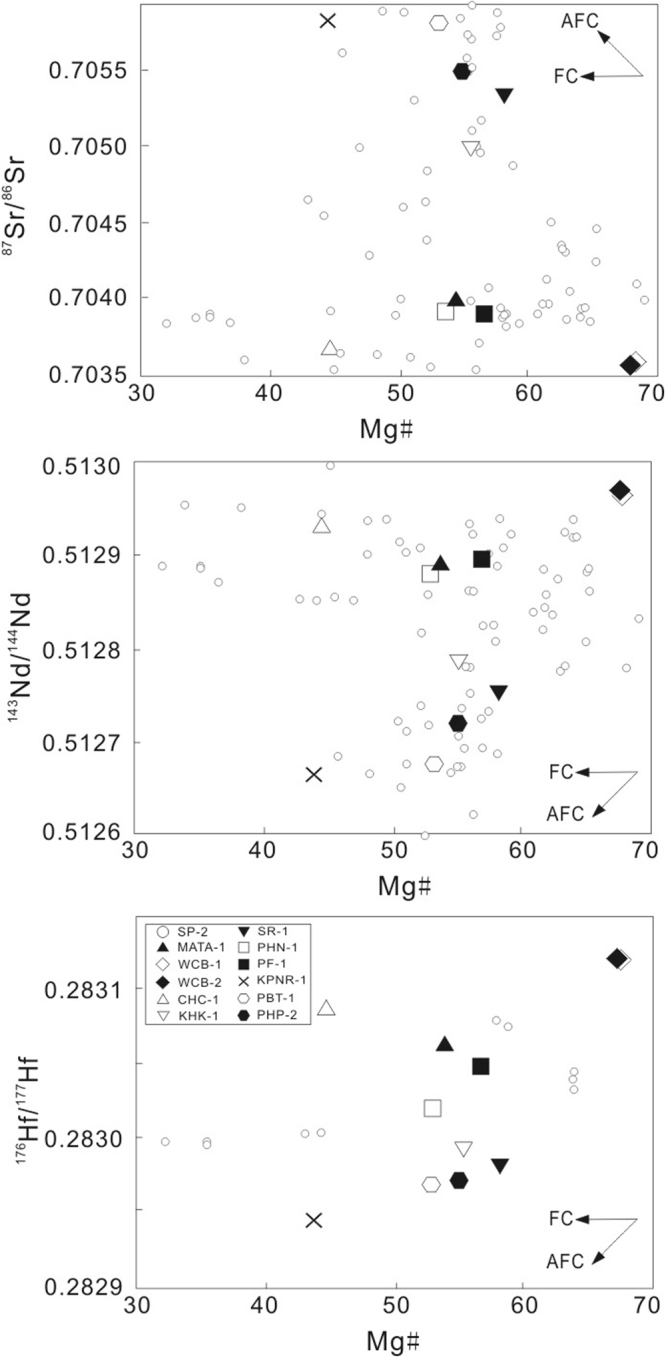
Mg# vs. ^87^Sr/^86^Sr (**a**), ^143^Nd/^144^Nd (**b**), and ^176^Hf/^177^Hf (**c**) isotopic ratios for late Cenozoic volcanic rocks from Thailand. Abbreviations: AFC = assimilation and fractional crystallization, and the possible contaminants is continental crust; FC = fractional crystallization. Symbols and data sources for Indochina block are the same as those in Fig. 6.

**Figure 9:**
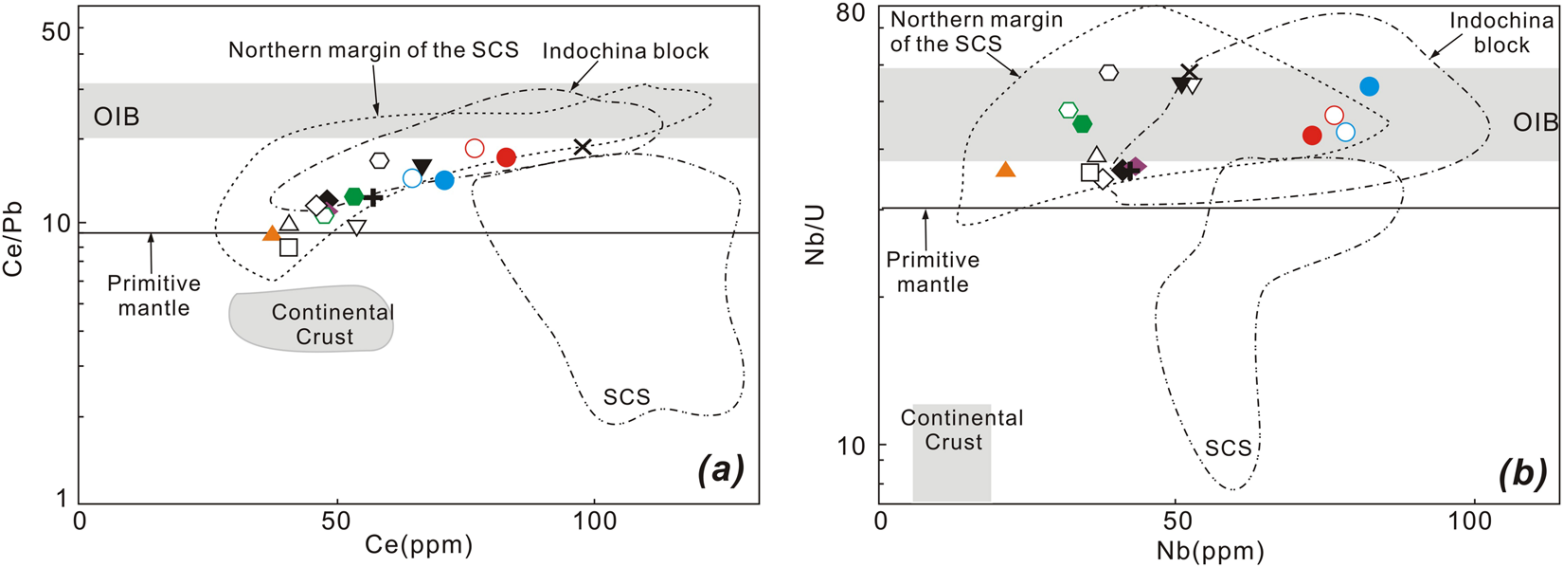
Plots of Ce/Pb vs. Ce (**a**) and Nb/U vs. N**b (b**) for the late Cenozoic volcanic rocks from Thailand. Symbols are the same as those in Fig. 3. Data for primitive mantle and oceanic island basalt (OIB) are from Hofmann^55^, and data for continental crust (CC) is from Rudnick and Gao^91^. Other data sources are the same as Fig. 6. Values in Y-axis is logarithmic scale.

**Figure 10:**
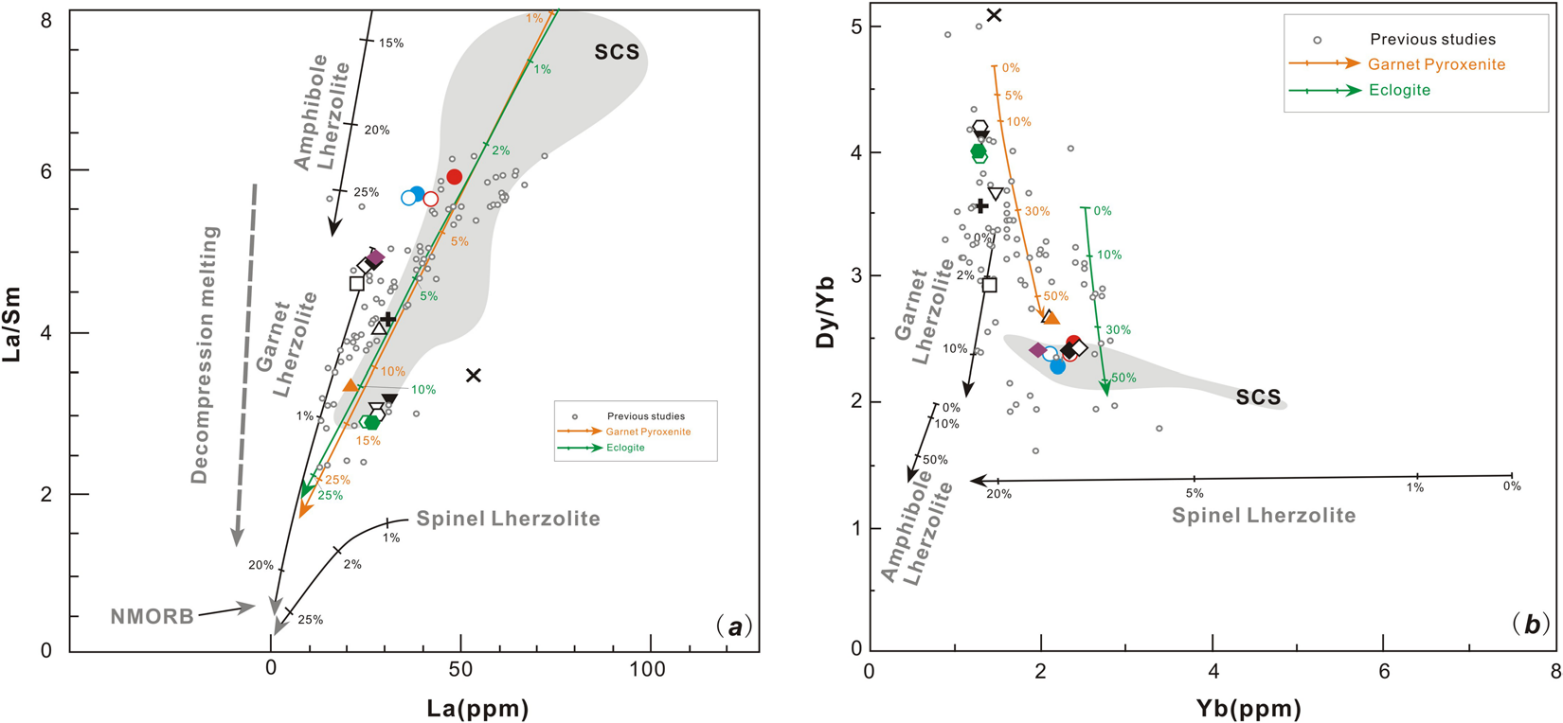
Partial melting (non-modal) models for late Cenozoic volcanic rocks from Thailand using light- and high rare earth elements. (**a**) La (ppm) versus La/Sm(ratio) and (**b**) Yb(ppm) versus Dy/Yb (ratio) diagrams that schematically illustrate the effect of variations in the degree of decompression partial melting of different mantle sources on the composition of mantle melts. Curves with tick marks represent equilibrium batch partial melting of an olivine:orthopyroxene: clinopyroxene:spinel (54:27:13:6 mixture) lherzolite, an amphibole-rich lherzolite (55:20:05:15:05 amphibole:olivine:orthopyroxene:clinopyroxene:garnet), a garnet pyroxenite (50:45:5 garnet:clinopyroxene:orthopyroxene), an eclogite (75:25 garnet: clinopyroxene), and a garnet lherzolite (olivine:orthopyroxene:clinopyroxene:garnet 55:25:15:5; percent at each tick mark represents degree of melting. For melting calculations the following parameters were used: bulk D for La was 0.0015, for Sm was 0.0406, for Dy was 0.050 and for Yb was 0.051 in the spinel lherzolite; bulk D for La was 0.089, for Sm was 0.579, for Dy was 0.354 and for Yb was 0.496 in the amphibole-lherzolite; bulk D for La was 0.0246, for Sm was 0.236, for Dy was 0.680 and for Yb was 2.143 in the garnet pyroxenite; bulk D for La was 0.0407, for Sm was 0.257, for Dy was 0.513 and for Yb was 1.218 in the eclogite; bulk D for La was 0.009, for Sm was 0.053, for Dy was 0.109 and for Yb was 0.256 in the garnet lherzolite. Symbols are the same as those in Fig. 3, and data sources are the same as those in Fig. 6.

Because the primary mantle magmas of Thailand volcanic rocks must pass through the continental crust before erupting on the surface, crustal contamination may play a significant role in their petrogenesis. Intraplate volcanic centers are widely dispersed within the Indochina block (Fig. 2), and we have collated the most recent data for these intraplate volcanic rocks from the literature^20–25^. In Figs 8 and 9, our data overlap with previously published data. There is no positive correlation between Mg# and ^87^Sr/^86^Sr or negative correlation between Mg# and ^143^Nd/^144^Nd or ^176^Hf/^177^Hf, which suggests a minimal role of continental contamination. Furthermore, the trace element ratios of these samples do not reflect any effects of continental contamination (Fig. 10). For example, Nb/Ta ratios (5.3–18.8, with average value of 14.9) and Zr/Hf ratios (37.8–45.8, with average value of 41.3) are close to primitive mantle (Nb/Ta = 17.5 ± 0.5 and Zr/Hf = 36.27)^59^, and obviously higher than those of continental crust^60^. In addition, their Ce/Pb ratios (7.6–18.5, with an average value of 12.2), and Nb/U ratios (33.8–52.6, with average value of 44.5) are mostly higher than those of primitive mantle (Ce/Pb = 9 and Nb/U ≈ 30)^59^ and close to those of oceanic basalts^53,54,59,61^. In Fig. 10, our new data plot outside the field of continental crust but within the field of basalts from the Indochina block and northern margin of the South China Sea^29^, that are bracketed by primitive mantle and oceanic island basalt compositions. Finally, our new data, together with other published data, plot within the field defined by oceanic island basalts (OIBs) in Sr-Nd-Hf-Pb isotope diagrams (Figs 6 and 7). These characteristics suggest that continental crustal contamination has been minimal during the genesis of intraplate volcanism in the wider South China Sea region, as previously suggested for the more restricted SCS basin^14,27,29,30^, Hainan Island^9,10^ and the Indochina block^23,25^. Some authors have however argued for a significant role of crustal contamination for Vietnamese basalts^20,24^.

#### Fractional crystallization

Compared to peridotite mantle, low content of some compatible elements (Supplementary Dataset Table 1) and plots of Mg# versus other oxides, CaO/Al_2_O_3_ and trace elements (Sc, Cr) (Fig. 4), all show that the parent magma (derived from a mantle source) for late Cenozoic basaltic lavas in Thailand may have undergone fractional crystallization of mafic minerals (e.g., olivine and clinopyroxene, etc) en route to the surface. For the younger group, with decreasing Mg#, SiO_2_, FeO^t^, Al_2_O_3_ and TiO_2_ decrease, CaO/Al_2_O_3_, Sc and Cr increase, and no systematic variations occur in CaO, Na_2_O and K_2_O (Fig. 4). These trends are consistent with a significant role of olivine + clinopyroxene crystallization in the magma evolution. Note that differences among samples from the same basaltic flow (e.g., Na Khon Ratchasima) may result from the variability of the parental magmas (Fig. 4). In addition, due to their highest content of MgO (and Mg# value), the older group (Wichian Buri basalts) with the oldest ages in this study can be regarded as relatively primitive magmas close to primary melts^51,52^.

#### Mantle end-members

As shown in Fig. 6a, Sr and Nd isotopic compositions for late Cenozoic volcanic rocks from the Indochina peninsula^20–25^ show a larger variation than those from the SCS basin^27–30^ and the northern margin of the SCS^5–14,29^, indicating that the mantle source beneath the former is more heterogeneous than those beneath the latter two. In Figs 6 and 7, Thailand basaltic rocks define a mixing trend between a depleted mantle end-member and a Samoa-like enriched mantle component (EMII), which is consistent with late Cenozoic volcanic rocks from the SCS basin and the northern margin of the SCS (Figs 6 and 7). In detail, except for these two younger samples (MATA-1 and CHC-1) that tap a local origin, the older samples with ages >2.3 Ma generally show more radiogenic Hf and Nd and less radiogenic Sr isotope compositions than the younger samples (0.6–0.9 Ma) (Supplementary Dataset Table 2), which indicates that the mantle origin of basaltic rocks in Thailand may have evolved with time. As modelled in Fig. 7a, the older group have more depleted mantle end member compositions than that of the younger group, which suggests that the mantle beneath Thailand became more and more enriched with time. In general, the above characteristics show that the mantle source for the late Cenozoic volcanic rocks from the SCS region can be explained by a simple binary mixing model^7–9,14,20–25,27–30^. However, what are the depleted and enriched mantle end-members for the late Cenozoic volcanic rocks of Thailand?

For the depleted mantle end-member of the late Cenozoic volcanic rocks from Thailand, many scientists have proposed that it should be an Indian ocean-type mantle^20–25^, as shown on Figs 6 and 7, because the Indian ocean-type mantle is prevalent in late Cenozoic intraplate volcanism in the southeast Asian^62^ and the SCS region^7,28–30^, and even widely distributed beneath the whole west Pacific region^63,64^. For the enriched mantle end-member, it may be EMII (enriched mantle type II), although some scientists have proposed that EM1 showing a DUPAL Pb anomaly may be involved in the origin of a small amount of late Cenozoic volcanic rocks in southern Vietnam^20,22^. Many scientists have proposed the involvement of EMII to explain the origin of post-spreading volcanic rocks from the SCS basin^28–30^, the northern margin of the SCS^10–14^, and southern Vietnam^25^ but the origin of EMII remains unclear.

Some scientists have proposed that the origin of EMII for late Cenozoic volcanic rocks of the Indochina block may be sub-continental lithospheric mantle (SCLM)^20,23^. However, SCLM, as the origin of EMII in this study, can be ruled out. Firstly, a significant Nd–Hf isotopic decoupling (resulting from fluid-driven metasomatism) can be observed for samples from the lithospheric mantle^23^, and yet late Cenozoic volcanic rocks from Thailand lie along the Terrestrial array (Fig. 6b). Secondly, the SCLM generally shows different Hf-Nd isotopic compositions from oceanic basalts (MORB + OIB)^23^, and late Cenozoic volcanic rocks from Thailand plot within the field of the latter (Fig. 6b). Thirdly, late Cenozoic volcanic rocks from Thailand shoe a positive Nb-Ta anomaly (Fig. 5), and yet basaltic rocks derived from SCLM generally exhibited negative anomalies in Nb and Ta (e.g., An *et al*.^25^). Thus, EMII did not originated from sub-continental lithospheric mantle (SCLM)^20,23^, but possibly from the Hainan mantle plume (see discussion below).

#### Mantle lithology and partial melting

It is important to consider lithological variations in the mantle source when trying to understand major- minor-, trace-element and isotopic compositions of basaltic rocks with no continental crustal contamination^65–67^. Partial melting experiments have shown that compositions equivalent to alkali basaltic magmas can be produced by melting garnet pyroxenite^68–70^, carbonated peridotite^71,72^, or eclogite^73,74^ +CO_2_^75^, and a mixture of these materials^76^. We have modeled the mantle lithology of late Cenozoic alkali volcanic rocks from Thailand, which are shown in Fig. 10. In addition, a batch partial melting model of garnet pyroxenite (50:45:5 garnet:clinopyroxene:orthopyroxene) alone can explain the genesis of late Cenozoic alkali volcanic rocks from Thailand (Fig. 10a,b). Relative to heavy rare earth elements (HREEs) only susceptible to melting garnet mineral in mantle source rock, light rare earth elements (LREEs) more likely reflect the extent of low degree partial melting. Additionally, we can conclude that late alkali Cenozoic volcanic rocks from Thailand can be produced by less than 15% partial melting as shown in Fig. 10a,. This result, combined with experimental petrologic data, imply that the mantle lithology of late Cenozoic alkali volcanic rocks in Thailand may be garnet pyroxenite^68^ already metasomatized by carbonaceous fluids (released from ancient recycled oceanic crust).

In addition, low contents of some compatible elements (Supplementary Dataset Table 1) and plots of Mg# versus other oxides, CaO/Al_2_O_3_ and trace elements (Sc, Cr) (Fig. 4), all show that parent magma derived from mantle source may undergo fractional crystallization of mafic minerals (e.g., olivine and clinopyroxene, etc) en route to the surface.

### Tectonic significance

#### Tectonic setting of late Cenozoic volcanic rocks from Thailand

**Figure 11:**
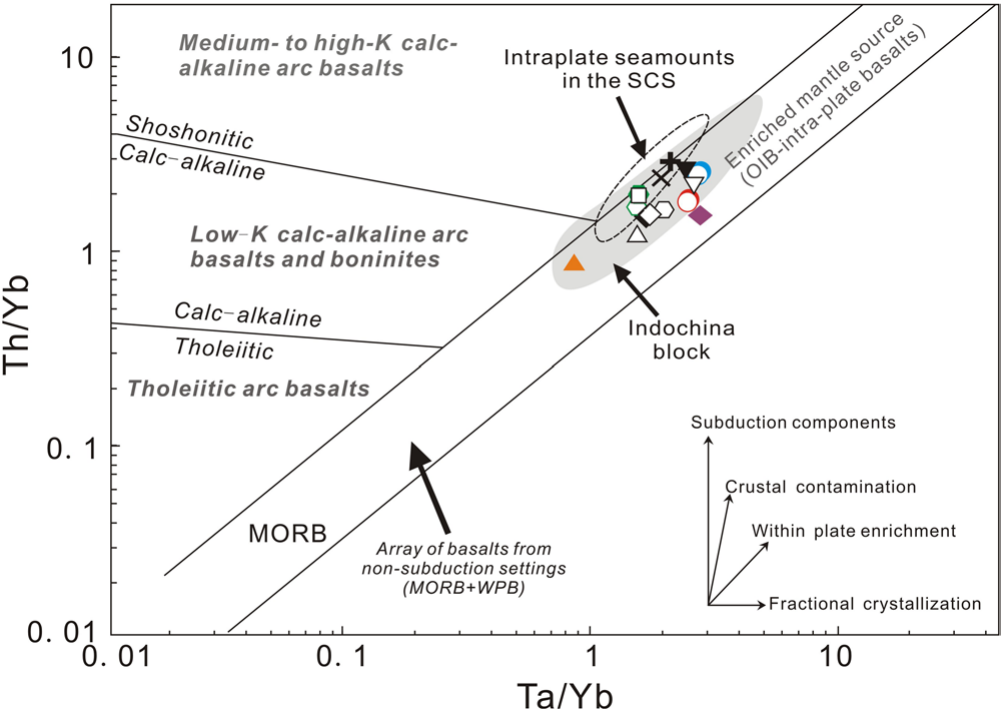
Trace element discrimination diagrams of Th/Yb versus Ta/Yb for late Cenozoic volcanic rocks from Thailand^77^. Values in X- and Y-axis are logarithmic scale. Symbols and data sources for Indochina block are the same as those in Fig. 3.

Except for those samples within or around the Khorat plateau that belong to intraplate basalts^23^ (Fig. 2), those samples from the Paleozoic Sukhothai arc terrane between CCS (Chiangmai-Changthaburi paleo-Tethys suture) and NUSKS, Nan-Uttaradit Sra Kaeo paleo-Tethys suture^39^, are close to the subduction zone formed by underthrustng of the Indian plate beneath the Eurasian plate^36^. Thus, the tectonic setting for those samples (Mae Tha and Sop Prop) needs to be further constrained. In the plot of Th/Yb versus Ta/Yb for discriminating tectonic setting of basaltic rocks^77^, all samples plot in the array of basalts from non-subduction settings (e.g., MORB, and within plate basalts) and lies close to enriched mantle source (OIB-intra-plate basalts) (Fig. 11), which is consistent with previous studies for basalts from the Indochina block and northern margin of the SCS, and post-spreading, intra-plate seamounts in the SCS^20–25,27,29,30^. The above characteristics, combined with major- and trace element and isotopic characteristics (Figs 3, 5 and 6), imply that most of samples are related to intraplate magmatism (mantle plume? See disscussions below).

#### Implications for deep mantle geodynamics process: Hainan plume

The South China Sea (SCS) region located in the convergence zone between the Eurasian plate, Indo-Australian plate and Philippine Sea plate (Pacific plate). The integrated effects from the India-Asian collision, eastward rollback of the Pacific subduction zone and northern migration of the Philippine Sea Plate affect Cenozoic geological evolution of the SCS region and Southeast Asia, e.g., the closure of the Proto-SCS and the opening of SCS in the period 32–16 Ma. After cessation of SCS spreading, large-scale volcanism occurred in the SCS (Fig. 1). The question then arises, what was the deep geodynamic process for the volcanism, plate tectonics or mantle plume?

Recent geophysical studies have shown that a mantle plume existed beneath Hainan Island^78–85^. Seismic tomographic data indicate that a sub-vertical low-velocity column is imaged beneath Hainan Island and the South China Sea and extends from shallow depths down to the 660-km discontinuity^79,80^, and even down to a depth of 1300 km^78,81–85^, and Montelli *et al*.^81^, based on global tomographic modeling, further suggested a broad low-velocity anomaly can extend down to 1900 km depth. In addition, several geophysical studies have suggested that the Hainan mantle may have originated from the core–mantle boundary^83–85^.

The existence of a Hainan plume has recently received support from incresing petrological and geochemical evidence. Based on calculation results for mantle potential temperatures beneath the SCS and geochemical studies on intraplate seamount basalts from the SCS, Yan and Shi^34^ and Yan *et al*.^27^ for the first time provided direct geological evidence for the presence of a Hainan plume as revealed by geophysical data. ^230^Th excesses in Hainan lavas imply a slowly (<1 cm/year) rising mantle plume^9^, as Montelli *et al*.^81^ suggested that it is a dying plume. Wang *et al*.^10,11^ also suggested that mantle potential temperatures beneath Hainan Island could be related to a Hainan plume. Yan *et al*.^29^ compiled petrological and geochemical data from the northern margin of the SCS, the SCS itself, and the Indochina block, and proposed that most late Cenozoic basaltic rocks from the region need an enriched end-member in the mantle source, implying the existence of a mantle plume (i.e., Hainan plume) in the SCS region. Yan *et al*.^29^ also further suggested that the plume may play a significant role on the overall Cenozoic tectonic evolution of the SCS, e.g., earlier rifted stage, subsequent seafloor spreading and later post-spreading volcanism. Details for the plume (including its duration, rifts (ridge) –plume interaction mechanism, etc) still need to be further clarified.

In particular, the question arises as to what is the western extent of the influence of the Hainan mantle plume? Considering the extensive occurrence of basaltic lava flows in SE Vietnam, Maruyama^86^ first proposed the idea that a Vietnamese mantle plume existed beneath Southeast Asia, and that it appeared to be verified by geological and geophysical data^87^. However, many geophysicists recently challenged the above assertion and pointed out that the low velocity anomaly from the Hainan mantle may extend to southern Vietnam^81,82,85^. Geochemically, Yan *et al*.^29^ suggested that late Cenozoic volcanic rocks in the Indochina block are genetically linked to the Hainan mantle plume, which is supported by recent studies of Vietnamese basalts^25^. Major-, and trace element compositions, and Sr-Nd-Pb-Hf isotopic ratios for Thailand basalts in this study indicates that they may have originated from the Hainan mantle plume. In addition, the western extent of the influence of the Hainan mantle plume may reach close to part of the Chiangmai-Changthaburi paleo-Tethys suture (North of the Cenozoic Mae Ping fault) in the Paleozoic Sukhothai arc terrane (Fig. 1). We envisge a tectonic scenario for the Hainan mantle plume as similar to the model depicted by Kincaid *et al*.^88^, i.e., a plume that ascends to the bottom of the lithosphere and then migrates along sloping rheologic boundary layers to lithospheric faults under extensional settings (e.g., reactivated paleo-sutures, spreading centers), eroding the lithosphere on its way upward (i.e., lithosphere/plume interaction) followed by eruptions at the surface^25,29,30^.

## Conclusions

In this study, we present new Hf isotope ratios, and major- and trace element concentrations, and Sr-Nd-Pb-Hf isotopic compositions of late Cenozoic basaltic lavas from Thailand. We suggested that,

1. Cenozoic basaltic lavas in Thailand are alkaline basaltic rocks and belong to a wider region of post-spreading intraplate magmatism in the SCS region.
2. Geochemically the alkaline basalts are oceanic island basalt (OIB)-like (e.g., enriched in mostly large-ion lithophile elements-LILEs and high field strength elements-HFSEs).
3. Sr-Nd-Hf-Pb isotopic compositions lay between DMM (depleted mid-ocean ridge basalt mantle) or Indian ocean-type mantle and EMII (enriched mantle type II) and imply that basalt origin can be explained by a simple binary mixture of these two mantle end-members.
4. The, EMII may have originated from the Hainan mantle plume.
5. Trace element partial melting modeling indicates that the alkaline basalts could have been produced by partial melting of garnet pyroxenite.
6. Post-spreading intraplate volcanism (induced by the Hainan mantle plume) in the SCS region extended westwards to affect the Paleozoic Sukhothai arc terrane between the Chiangmai-Changthaburi Paleo-Tethys suture and the Nan-Uttaradit Sra Kaeo suture.

## Electronic supplementary material

Dataset 1

Dataset 2
